# The effect of long-term poor sleep quality on risk of back-related disability and the modifying role of physical activity

**DOI:** 10.1038/s41598-021-94845-7

**Published:** 2021-07-28

**Authors:** Eivind Schjelderup Skarpsno, Tom Ivar Lund Nilsen, Paul Jarle Mork

**Affiliations:** 1 Department of Public Health and Nursing, Norwegian University of Science and Technology (NTNU), 7491 Trondheim, Norway; 2 Department of Neurology and Clinical Neurophysiology, St. Olavs Hospital, Trondheim, Norway; 3 Clinic of Anaesthesia and Intensive Care, St. Olavs Hospital, Trondheim University Hospital, Trondheim, Norway

**Keywords:** Pain, Chronic pain, Risk factors, Signs and symptoms, Medical research, Epidemiology

## Abstract

Sleep problems and regular leisure time physical activity (LTPA) are interrelated and have contrasting effects on risk of back pain. However, no studies have investigated the influence of long-term poor sleep quality on risk of back-related disability, or if LTPA modifies this association. The study comprised data on 8601 people who participated in three consecutive surveys over ~ 22 years, and who reported no chronic back pain at the two first surveys. Adjusted risk ratios (RRs) for back-related disability were calculated at the last survey, associated with the joint effect of changes in sleep quality between the two first surveys and meeting physical activity guidelines at the second survey. Compared to people with long-term good sleep, people with long-term poor sleep had nearly twice the risk of back-related disability (RR 1.92, 95% CI 1.48–2.49). There was no statistical interaction between sleep and LTPA but people who reported long-term poor sleep and meeting the physical activity guidelines had 35% lower risk of back-related disability compared to people with same level of sleep problems, but who not met the guidelines. These findings suggest that long-term poor sleep quality contributes to a substantially increased risk of chronic and disabling back pain irrespective of LTPA.

## Introduction

Back pain, defined as pain anywhere along the spine, from the neck down to the hips, is the worldwide leading cause of years lived with disability^1,2^. In addition to the suffering of affected individuals, back pain is among the main causes of sick leave and early retirement^3^. The negative impact of disabling back pain, both on the individual and the society, underscores the importance of identifying modifiable health behaviors that can be targeted for interventions and preventive measures.

It is well established that sleep problems are associated with increased risk of chronic back pain^4–8^ as well as poor prognosis or exacerbation of symptoms in pain-afflicted individuals^9–11^. Conversely, leisure time physical activity has been associated with multiple health benefits^12–15^, including reduced risk of back pain^16–18^. There is also evidence indicating that regular physical activity improves sleep quality^19–22^, which in turn may reduce risk of other adverse health outcome^23,24^. Sleep problems and regular leisure time physical activity therefore appear to be interrelated as well as having contrasting effects on risk of back pain. This notion is in part supported by some previous studies, showing that leisure time physical activity may attenuate the adverse effect of poor sleep on risk of chronic back pain^6^ and fibromyalgia^25^. However, there is a paucity of studies investigating the influence of long-term changes in sleep quality on risk of chronic back pain and back-related disability and if leisure time physical activity modifies this association. Studying long-term changes in sleep quality and the modifying role of leisure time physical activity may provide further insight into the risk-enhancing and risk-reducing effects of poor sleep and physical activity.

The aim of this study was therefore to examine the association between long-term poor sleep quality and risk of chronic back pain and back-related disability, and if meeting physical activity guidelines modify these associations.

## Methods

### Study population

All inhabitants aged 20 years or older in Trøndelag County, Norway, were invited to participate in four health surveys (the HUNT Study), first in 1984–1986, then in 1995–1997 and 2006–2008, and last in 2017–2019. Information about chronic musculoskeletal pain was not collected in 1984–1986 and the current study is therefore based on data from 25,909 participants who participated in 1995–1997, 2006–2008, and 2017–2019. Of these, 8601 persons did not report chronic back pain in 1995–1997 and 2006–2008 and responded to pain questions in 2017–2019. To improve efficiency and reduce potential bias due to missing data, a simulation-based multiple imputation procedure was used to replace missing observations on changes in sleep quality (n = 836, 9.7%) and physical activity (n = 140, 1.6%) in 2006–2008. Further information regarding the HUNT Study can be found elsewhere^26^.

The study was approved by the Regional Committee for Ethics in Medical Research (project no. 2014/612 REK midt) and all participants gave informed written consent allowing the use of their data and samples for research.

### Sleep quality in 1995–1997 and 2006–2008

Sleep quality in the 1995–1997 survey was assessed by three questions: (1) ‘How often during the last month have you had difficulty falling asleep at night?’, (2) ‘How often during the last month have you woken too early and couldn’t get back to sleep?’, and (3) ‘How often do you suffer from sleeplessness?’. The two first questions had the response options ‘never’, ‘occasionally’, ‘often’, and ‘almost every night’. The last question had the response options ‘never, or just a few times a year’, ‘1–2 times a month’, ‘approximately once a week’ and ‘more than once a week’. Participants were classified with ‘poor sleep quality’ if they answered ‘often/almost every night’ on the questions on persistent sleep difficulty (‘difficulty falling asleep at night’ and/or ‘waking up too early’) and/or ‘more than once a week’ on the question on sleeplessness. Participants who answered ‘never/occasionally’ on the questions on persistent sleep difficulty and sleeplessness less than once a week were classified as having ‘good sleep quality’.

Sleep quality in the 2006–2008 survey was also assessed by three questions: (1) ‘How often during the last three months have you had difficulty falling asleep at night?’, (2) ‘How often during the last three months have you woken too early and couldn’t get back to sleep?’, and (3) ‘How often during the last three months have you woken up repeatedly during the night?’. Each question had three response options (i.e., ‘never/seldom’, ‘sometimes’, and ‘several times a week’). Participants were classified as having ‘poor sleep quality’ if they answered ‘several times a week’ on at least one of the questions. Participants who answered ‘never/seldom/sometimes’ on these questions were classified as having ‘good sleep quality’.

The information on sleep quality from the two first surveys (1995–1997 and 2006–2008) was then used to categorize the participants into one of four groups: (1) ‘long-term good sleep’, (2) ‘poor sleep to good sleep’, (3) ‘good sleep to poor sleep’, and (4) ‘long-term poor sleep’.

### Leisure time physical activity in 2006–2008

Leisure time physical activity was assessed by the following question: ‘How much of your leisure time have you been physically active during the last year (please include active commuting)’. When responding to this question, the participants were asked to specify a weekly average of number of hours of light (no sweating or heavy breathing) and/or hard (sweating and heavy breathing) physical activity with the response options: ‘none’, ‘less than 1 h’, ‘1–2 h’, and ‘3 or more hours’ separately for light and hard activity. Participants were categorized into two levels of leisure time physical activity (i.e., ‘meeting physical activity guidelines’ and ‘not meeting physical activity guidelines’) according to the guidelines for physical activity among adults at the time of the 2006–2008 survey^27^, i.e., ≥ 30 min on 5 days each week for moderate-intensity aerobic physical activity or ≥ 20 min on 3 days per week for vigorous-intensity aerobic physical activity. Accumulation of ≥ 150 min/week of moderate-to-vigorous physical activity or ≥ 60 min/week of vigorous physical activity was defined as ‘meeting physical activity guidelines’ while not achieving these recommendations was defined as ‘not meeting physical activity guidelines’. A combination of moderate- and vigorous physical activity was defined as ‘meeting physical activity guidelines’ if fulfilling the minimum level of the recommendations.

### Back pain in 1995–1997, 2006–2008 and 2017–2019

Questions on chronic musculoskeletal pain were adopted from the Standardized Nordic Questionnaire^28^, which has been shown to have acceptable reliability and validity^29,30^. At the two first surveys in 1995–1997 and 2006–2008, all participants were asked ‘During the last year, have you had pain and/or stiffness in your muscles and joints that lasted for at least three consecutive months?’, with the response options ‘no’ and ‘yes’. Participants answering ‘yes’ were asked to indicate the affected body area(s), including neck, shoulders, upper back, elbows, low back, hips, wrists/hand, knees, and ankles/feet. Participants who reported to have pain in the neck, upper back, or low back pain in 1995–1997 and 2006–2008 were excluded from the study.

At follow-up in 2017–2019, all participants were asked ‘During the last year, have you had pain in your muscles and joints that lasted for at least three consecutive months?’. Participants who answered ‘yes’ to this question were asked to indicate the affected body area(s), including neck, shoulders, upper back, elbows, low back, hips, wrists/hand, knees, and ankles/feet. Those who reported pain in the neck, upper back, or low back were considered to have chronic back pain. Back-related disability was assessed in relation to work ability and ability to carry out leisure time activities. Participants were asked the following two questions: ‘Has the pain in muscles or joints reduced your ability to work?’, and ‘Has the pain in muscles or joints reduced your leisure activity?’, with the response options ‘no’ and ‘yes’. Participants were classified with back-related disability if they had chronic back pain and reported reduced work ability and/or reduced leisure time activity.

### Possible confounders

All potential confounders were assessed at the second survey in 2006–2008 except education (assessed in 1995–1997) and change in body weight from 1995–1997 to 2006–2008. Educational level was categorized into ‘primary school’, ‘high school’, ‘college ≤ 4 years’ and ‘college > 4 years’. Measurements of height and weight obtained at the clinical examinations were used to calculate body mass index (BMI) as weight divided by the square of height (kg/m^2^)^31^. Relative change in body weight was calculated as percentage change between the 1995–1997 survey and the 2006–2008 survey. Smoking status was divided into ‘never smoked’, ‘former smoker’, ‘current low-intensity smoker (< 10 cigarettes per day)’, ‘medium-intensity smoker (10–19 cigarettes per day)’ and ‘high-intensity smoker (20 or more cigarettes per day)’. Symptoms of anxiety and depression were assessed by the Hospital Anxiety and Depression Scale (HADS) with a cut-off score of ≥ 8 on both anxiety and depression^32^. To assess comorbid conditions, participants were asked if they have or have had heart disease, lung diseases, diabetes, or cancer. Presence of sleep apnea and restless legs was assessed by questions on breathing pauses and/or tingling in legs during sleep or in relation to sleep’. Physical work demands were defined based on self-reported description of work demands (‘mostly sedentary’, ‘much walking, ‘much walking or lifting’, and ‘heavy physical work’) and shift work was divided into ‘unemployed/not working’, ‘no’, and ‘yes’. Self-reported use of pain medication was assessed in both 1995–1997 and 2006–2008.

### Statistical analyses

A modified Poisson regression model was used to estimate risk ratios (RRs) for chronic back pain and back-related disability at follow-up in 2017–2019 associated with long-term changes in sleep quality between the two first surveys (1995–1997 and 2006–2008) and the joint effect of changes in sleep quality and meeting physical activity guidelines in 2006–2008. People who reported long-term poor sleep or changed sleep quality in this period were compared with the reference group of people with long-term good sleep. For the analysis of joint effect between long-term poor sleep quality and physical activity, people who reported good sleep in 1995–1997 and 2006–2008, and who met physical activity guidelines in 2006–2008 served as reference group. The precision of the RRs was assessed by 95% confidence intervals (CIs) using robust variance estimation. All associations were adjusted for age, sex, education, BMI, smoking, and percentage change in body mass. Missing data on education, BMI, relative change in body mass, and smoking were imputed. Potential effect modification between changes in sleep quality and adherence to physical activity recommendations was assessed as departure from additive effects calculating the relative excess risk due to interaction (RERI) with 95% CIs^33^.

Four supplementary analyses were conducted to assess the robustness of the results. First, to assess the potential influence of reverse causation, i.e., that people with acute pain reported poor sleep or less physical activity, the main analysis were repeated excluding people who reported use of pain medication. Second, both sleep apnea and restless legs syndrome are associated with chronic pain^34,35^, and the main analysis were therefore repeated excluding people reporting these specific sleep disorders. Third, since physical work demands are associated with chronic pain^36^ and higher prevalence of sleep problems^37^, the main analysis were repeated adjusting for shift work and occupational physical activity. Finally, there is an overlap but unclear temporal association between sleep quality and comorbid conditions^38–40^. Since anxiety and/or depression and other comorbid conditions (e.g., heart disease, lung diseases, diabetes, cancer) may act both as mediators and confounders to the association between poor sleep and chronic back pain, these factors were excluded from the main analyses and instead adjusted for them in sensitivity analyses.

All statistical analyses were performed using Stata for Windows, version 16.0 (StataCorp LP, College Station, Texas).

### Ethical approval

The study was approved by the Regional Committee for Ethics in Medical Research (project no. 2014/612 REK midt). The study was carried out according to the Declaration of Helsinki.

## Results

Among the 8601 participants without chronic back pain in 1995–1997 and 2006–2008, 20.0% (1727) and 8.5% (733) reported chronic back pain or back-related disability at follow-up in 2017–2019. Table 1 shows the characteristics of the study population stratified by change in sleep quality from 1995–1997 to 2006–2008.

**Table 1:** Characteristics of the study population stratified by change in sleep quality from 1995–1997 to 2006–2008.

	Change in sleep quality from 1995–1997 to 2006–2008			
	Remained good sleep	Poor sleep to good sleep	Good sleep to poor sleep	Remained poor sleep
Participants, no. (%)	6636 (77.2)	433 (5.0)	1106 (12.9)	426 (5.0)
Age, mean (SD)	53.6 (11.4)	57.1 (12.6)	55.8 (11.1)	57.7 (11.7)
Females, no. (%)	3422 (51.6)	252 (58.2)	646 (58.4)	253 (59.4)
Higher education, no. (%)^a^	2111 (31.8)	116 (26.8)	355 (32.1)	128 (30.1)
Body mass index, mean (SD)	27.0 (3.9)	27.0 (4.1)	27.0 (4.0)	27.1 (4.1)
Percentage change in body mass, mean (SD)^b^	4.7 (8.0)	4.8 (8.4)	4.7 (8.4)	4.2 (9.3)
Current smoker, no. (%)	925 (13.9)	83 (19.1)	155 (14.0)	63 (14.8)
Shift work, no. (%)	1371 (20.7)	86 (19.9)	207 (18.7)	17.4 (74)
Anxiety and/or depression, no. (%)^c^	498 (7.5)	70 (16.2)	238 (21.5)	135 (31.7)
Comorbid condition(s), no. (%)	1173 (17.7)	105 (24.3)	268 (24.2)	104 (24.4)
Sleep apnea and/or restless legs syndrome, no. (%)	271 (4.1)	23 (5.3)	113 (10.2)	54 (12.7)

*SD* standard deviation.

^a^College or higher in 1995–1997.

^b^Percentage change from 1995–1997 to 2006–2008.

^c^Measured by Hospital Anxiety and Depression Scale score ≥ 8.

Table 2 shows the associations between long-term changes in sleep quality and risk of chronic back pain and back-related disability. Compared to participants who reported good sleep in both 1995–1997 and 2006–2008, the RRs for chronic back pain and back-related disability were 1.65 (95% CI 1.42–1.93) and 1.92 (95% CI 1.48–2.49) for those with long-term poor sleep, respectively.

**Table 2:** Risk of chronic back pain and back-related disability at follow-up in 2017–2019 associated with change in sleep quality from 1995–1997 to 2006–2008.

Risk of back pain and change in sleep quality from 1995–1997 to 2006–2008	No. of persons	No. of cases	Age-adjusted, RR^a^	Multi-adjusted RR (95% CI)^b^
**Chronic back pain**				
Remained good sleep	6636	1212	1.00	1.00 (reference)
Poor sleep to good sleep	433	101	1.35	1.30 (1.08–1.55)
Good sleep to poor sleep	1106	275	1.35	1.31 (1.16–1.48)
Remained poor sleep	426	129	1.71	1.65 (1.42–1.93)
**Back-related disability**				
Remained good sleep	6636	486	1.00	1.00 (reference)
Poor sleep to good sleep	433	50	1.65	1.55 (1.17–2.06)
Good sleep to poor sleep	1106	138	1.66	1.59 (1.32–1.92)
Remained poor sleep	426	59	2.01	1.92 (1.48–2.49)

*CI* confidence interval, *RR* risk ratio.

^a^Adjusted for age (continuous).

^b^Adjusted for age (continuous), sex (women, men), education (primary school, high school, college ≤ 4 years, college > 4 years), body mass index (continuous), relative change in body weight (continuous), changes in physical activity (remained active, inactive to active, active to inactive, remained inactive), and smoking (never smoked, former smoker, current low-intensity smoker [< 10 cigarettes per day], medium-intensity smoker [10–19 cigarettes per day] and high-intensity smoker [20 or more cigarettes per day]).

Table 3 shows the joint association between changes in sleep quality from 1995–1997 to 2006–2008 and meeting the physical activity guidelines in 2006–2008, and risk of chronic back pain and back-related disability, respectively. The reference group comprised people who reported good sleep in both 1995–1997 to 2006–2008 and met the physical activity guidelines in 2006–2008. Compared to the reference group, people with long-term poor sleep had a RR for chronic back pain of 1.51 (95% CI 1.18–1.94) if they met the physical activity guidelines and a RR of 1.69 (95% CI 1.38–2.08) if they did not meet the guidelines. People with long-term good sleep who did not meet the guidelines had a RR of 1.01 (95% CI 0.90–1.13). There was no clear evidence of a modifying effect of physical activity (RERI 0.17, 95% CI − 0.39 to 0.88). Corresponding RRs for back-related disability were 1.70 (95% CI 1.11–2.61), 2.05 (95% CI 1.42–2.96), and 1.10 (95% CI 0.91–1.33), respectively, with a RERI of 0.25 (95% CI − 0.47–1.05).

**Table 3:** Risk of chronic back pain and back-related disability at follow-up in 2017–2019 associated with changes in sleep quality from 1995–1997 to 2006–2008 and adherence to physical activity recommendations in 2006–2008.

Risk of back pain and change in sleep quality from 1995–1997 to 2006–2008	Meeting physical activity guidelines				Not meeting physical activity guidelines			
	No. of persons	No. of cases	Age-adjusted, RR^a^	Multi-adjusted RR (95% CI)^b^	No. of persons	No. of cases	Age-adjusted, RR^a^	Multi-adjusted RR (95% CI)^b^
**Chronic back pain**								
Remained good sleep	3486	636	1.00	1.00 (reference)	3004	568	1.08	1.01 (0.90–1.13)
Poor sleep to good sleep	207	50	1.35	1.33 (1.01–1.76)	270	66	1.36	1.21 (0.93–1.58)
Good sleep to poor sleep	557	130	1.30	1.27 (1.06–1.52)	614	146	1.44	1.31 (1.10–1.56)
Remained poor sleep	208	56	1.57	1.51 (1.18–1.94)	255	75	1.88	1.69 (1.38–2.08)
**Back-related disability**								
Remained good sleep	3486	242	1.00	1.00 (reference)	3004	239	1.19	1.10 (0.91–1.33)
Poor sleep to good sleep	207	23	1.71	1.70 (1.11–2.61)	270	34	1.86	1.61 (1.10–2.36)
Good sleep to poor sleep	557	62	1.63	1.58 (1.18–2.08)	614	69	1.81	1.60 (1.22–2.10)
Remained poor sleep	208	25	1.79	1.70 (1.11–2.61)	255	39	2.35	2.05 (1.42–2.96)

*CI* confidence interval, *RR* risk ratio.

^a^Adjusted for age (continuous).

^b^Adjusted for age (continuous), sex (women, men), education (primary school, high school, college ≤ 4 years, college > 4 years), body mass index (continuous), relative change in body weight (continuous), and smoking (never smoked, former smoker, current low-intensity smoker [< 10 cigarettes per day], medium-intensity smoker [10–19 cigarettes per day], high-intensity smoker [20 or more cigarettes per day]).

### Supplementary analyses

The estimated RRs remained similar when excluding people who used pain medication or who reported possible sleep apnea or restless legs syndrome 1995–1997 and/or 2006–2008 (Supplementary Tables 1 and 2). Likewise, additional adjustments for work conditions, comorbid conditions, and anxiety and/or depression, and had negligible influence on the estimated RRs (Supplementary Tables 3–5).

## Discussion

This large-scale population-based study indicates that long-term poor sleep contributes to a substantially increased risk of chronic and disabling back pain that is largely independent of adherence to the physical activity guidelines. However, meeting the physical activity guidelines may reduce the unfavorable effect of long-term poor sleep on the risk of chronic back pain.

Previous studies that have shown an association between poor sleep quality and increased risk of chronic back pain have not considered long-term changes in sleep quality^4–8^. The findings in the current study are therefore important as they show that poor sleep quality over ~ 10 years is associated with 65–92% greater risk of chronic back pain and back-related disability compared to good sleep quality over the same period. In comparison, people who improved, or worsened their sleep quality had ~ 30% and ~ 55% greater risk of chronic back pain and back-related disability, respectively. These findings show that long-term poor sleep quality represents an important risk factor for chronic back pain, and in particular back-related disability. Preventive measures and interventions targeting long-term poor sleep quality may therefore help reduce the burden of back-related disability. To fully understand the adverse effect of poor sleep quality, further research is needed to assess whether long-term variations of other sleep characteristics (e.g., sleep duration, different measures of sleep quality, and circadian preferences) are associated with risk of chronic back pain and back-related disability.

Although there was no evidence of statistical interaction between poor sleep and leisure time physical activity, people who reported long-term poor sleep and not meeting the physical activity guidelines had 18–35% increased risk of chronic back pain and back-related disability compared to people with same level of sleep problems, but who met the physical activity guidelines. These findings indicate that meeting the physical activity guidelines weakens the unfavorable effect of long-term poor sleep on risk of chronic and disabling back pain. The current study extends on previous findings, which have indicated that leisure time physical activity reduces the unfavorable effect of poor sleep on risk of widespread pain^25^ and chronic pain in the neck and low back^6^. However, none of these previous studies examined the influence of long-term poor sleep quality or quantified physical activity in relation to the guidelines. A possible explanation for the modifying effect of leisure time physical activity on the association between long-term poor sleep quality and risk of chronic back pain is that the anti-inflammatory effect of physical activity^41,42^ reduces inflammation induced by poor sleep quality or insufficient sleep duration^43,44^. This view is supported by studies showing that physical activity may reduce pain perception and lead to increased pain tolerance^45,46^.

There are several strengths to the current study, such as the large population-based study sample, the prospective design, the assessment of long-term changes in sleep quality, and the detailed information on possible confounders. However, some limitations should be considered in the interpretation of the results. First, incident cases of chronic back pain and back-related disability at the follow-up in 2017–2019 were assessed among people who were able to and chose to participate in all three surveys. Thus, if people with poor sleep or who were physically inactive in 1995–1997 or 2006–2008 were less likely to participate at follow-up in 2017–2019, the estimated RRs may be underestimated. Second, misclassification of sleep and leisure time physical activity cannot be ruled out, i.e., the questionnaire-based nature of the data allows for subjective interpretation of the questions and individual perception of sleep quality and leisure time physical activity. Thus, objective measures that capture information about activity type context (e.g., work versus leisure), intensity, and duration, will likely improve the knowledge about the interplay between sleep quality and physical activity behavior. It should be noted that the current study did not assess the potential modifying effect of occupational physical activity or sedentary behavior. Fourth, guidelines for physical activity among adults at the time of the survey used in the current study (2006–2008)^27^ differ from the current guidelines^47^. However, due to the nature of the physical activity questions in the current study, it was difficult to differentiate between the former physical activity guidelines and the up-to-date guidelines (i.e., 150–300 min/week of moderate-intensity, or 75–150 min/week of vigorous-intensity physical activity, or comparable combination of moderate and vigorous-intensity physical activity)^47^. However, the key points regarding level of physical activity remain largely unchanged in the up-to-date guidelines and are therefore likely to induce negligible changes to the classification of participants. Fifth, sleep quality was assessed using different questions in 1995–1997 and 2006–2008, and there was no information about variations in sleep quality during the follow‐up or if new risk factors appeared. Sixth, biased estimates due to confounding by unmeasured or unknown factors cannot be ruled out in this type of study. For instance, it is possible that there exist common causes for both physical activity and chronic musculoskeletal pain, potentially opening a pathway from sleep to chronic pain when stratifying on physical activity. Although it is possible that the findings are influenced by reverse causation, the observed estimates remained similar when people reporting use of pain medication or physical pain the last month in the two first surveys were excluded. Finally, adjustments for variables associated with chronic back pain and back-related disability, such as genetic predisposition could be of importance^48^.

In conclusion, this large-scale population-based study shows that long-term poor sleep quality is associated with increased risk of chronic back pain and back-related disability irrespective of meeting the public health physical activity guidelines. However, meeting the physical activity guidelines may reduce the unfavorable effect that long-term poor sleep has on chronic back pain risk. These findings suggest that promoting good sleep and a physically active lifestyle throughout adulthood may have the potential to reduce the incidence of chronic back pain and back-related disability. However, this study highlights the need for high-quality studies with valid measurements of different sleep dimensions (sleep duration, sleep quality, circadian preferences) along with objectively measured physical activity to fully understand the interplay between sleep quality and physical activity behavior on risk of chronic and disabling back pain.

## Supplementary Information

Supplementary Tables.

## Data Availability

This study used data from the HUNT Study (https://www.ntnu.edu/hunt). Due to participant confidentiality, participant data is not publicly available. Any research group with a Principal Investigator affiliated with a Norwegian research institute can apply for access to use data from the HUNT Study. This means that researchers from non-Norwegian countries must have a collaboration partner in Norway to be able to use data from the HUNT Study. Each project needs to be approved by the HUNT Data Access Committee, Regional Medical Ethical Committee, in some cases also the Data Inspectorate.
