# Supplementary material for: The effect of long-term poor sleep quality on risk of back-related disability and the modifying role of physical activity
Source: Sci Rep. 2021 Jul 28;11:15386. doi: 10.1038/s41598-021-94845-7 (PMC8319142; doi:10.1038/s41598-021-94845-7)
Supplement: Supplementary file 1 — Supplementary Tables. [file 41598_2021_94845_MOESM1_ESM.pdf]

SUPPLEMENTARY MATERIAL

The effect of long-term poor sleep quality on risk of back-related disability and the modifying role of physical activity

Eivind Schjelderup Skarpsno[1,2*], Tom Ivar Lund Nilsen[1,3,] Paul Jarle Mork[1]

[1]Department of Public Health and Nursing, Norwegian University of Science and Technology (NTNU), Trondheim, Norway, [2]Department of Neurology and Clinical Neurophysiology, St. Olavs Hospital, Trondheim, Norway, [3]Clinic of Anaesthesia and Intensive Care, St Olavs Hospital, Trondheim University Hospital, Trondheim, Norway,

*Corresponding author: Eivind Schjelderup Skarpsno, PhD, Department of Public Health and Nursing, Norwegian University of Science and Technology (NTNU), 7491 Trondheim, Norway. E-mail: eivind.s.skarpsno@ntnu.no, phone: +47 97521297

Supplementary table 1. Risk of chronic back pain and back-related disability at follow-up in 2017-19 associated with changes in sleep quality from 1995-97 to 2006-08 and adherence to physical activity recommendations in 2006-08. People reporting use of pain medication in 1995-97 and/or 2006-08 were excluded from the analysis.

	Meeting physical activity guidelines				Not meeting physical activity guidelines			
Risk of back pain and change in sleep quality from 1995-97 to 2006-08	No. of persons	No. of cases	Age-adjusted, RR[a]	Multi-adjusted RR (95% CI)[b]	No. of persons	No. of cases	Age-adjusted, RR[a]	Multi-adjusted RR (95% CI)[b]
Chronic back pain								
Remained good sleep	2,742	474	1.00	1.00 (reference)	2,350	432	1.08	1.03 (0.91-1.17)
Poor sleep to good sleep	158	35	1.35	1.29 (0.93-1.79)	206	49	1.36	1.27 (0.94-1.70)
Good sleep to poor sleep	412	91	1.30	1.27 (1.03-1.57)	462	106	1.44	1.31 (1.07-1.60)
Remained poor sleep	148	35	1.57	1.40 (1.00-1.96)	182	50	1.88	1.66 (1.29-2.14)
Back-related disability								
Remained good sleep	2,742	178	1.00	1.00 (reference)	2,350	178	1.19	1.18 (0.95-1.46)
Poor sleep to good sleep	158	16	1.71	1.63 (0.96-2.76)	206	28	1.86	1.82 (1.18-2.82)
Good sleep to poor sleep	412	47	1.63	1.80 (1.31-2.49)	462	49	1.81	1.64 (1.18-2.28)
Remained poor sleep	148	17	1.79	1.79 (1.05-3.06)	182	28	2.35	2.18 (1.42-3.34)

Abbreviations: CI, confidence interval; RR, risk ratio

[a]Adjusted for age (continuous)

[b]Adjusted for age (continuous), sex (women, men), education (primary school, high school, college ≤4 years, college >4 years), body mass index (continuous), relative change in body weight (continuous), and smoking (never smoked, former smoker, current low-intensity smoker [<10 cigarettes per day], medium-intensity smoker [10-19 cigarettes per day], high-intensity smoker [20 or more cigarettes per day])

Supplementary table 2. Risk of chronic back pain and back-related disability at follow-up in 2017-19 associated with changes in sleep quality from 1995-97 to 2006-08 and adherence to physical activity recommendations in 2006-08. People reporting sleep apnea and/or restless legs syndrome in 1995-97 and/or 2006-08 were excluded from the analysis.

	Meeting physical activity guidelines				Not meeting physical activity guidelines			
Risk of back pain and change in sleep quality from 1995-97 to 2006-08	No. of persons	No. of cases	Age-adjusted, RR[a]	Multi-adjusted RR (95% CI)[b]	No. of persons	No. of cases	Age-adjusted, RR[a]	Multi-adjusted RR (95% CI)[b]
Chronic back pain								
Remained good sleep	3,363	597	1.00	1.00 (reference)	2,861	533	1.08	1.00 (0.90-1.12)
Poor sleep to good sleep	194	46	1.35	1.34 (1.00-1.78)	250	59	1.36	1.21 (0.92-1.59)
Good sleep to poor sleep	501	112	1.30	1.23 (1.01-1.49)	560	128	1.44	1.29 (1.07-1.55)
Remained poor sleep	187	50	1.57	1.53 (1.18-2.00)	224	62	1.88	1.60 (1.28-2.01)
Back-related disability								
Remained good sleep	3,486	229	1.00	1.00 (reference)	3,004	218	1.19	1.09 (0.90-1.32)
Poor sleep to good sleep	207	21	1.71	1.65 (1.06-2.60)	270	32	1.86	1.75 (1.20-2.57)
Good sleep to poor sleep	557	53	1.63	1.52 (1.12-2.06)	614	58	1.81	1.55 (1.15-2.10)
Remained poor sleep	208	19	1.79	1.48 (0.90-2.44)	255	30	2.35	1.80 (1.19-2.74)

Abbreviations: CI, confidence interval; RR, risk ratio

[a]Adjusted for age (continuous)

[b]Adjusted for age (continuous), sex (women, men), education (primary school, high school, college ≤4 years, college >4 years), body mass index (continuous), relative change in body weight (continuous), and smoking (never smoked, former smoker, current low-intensity smoker [<10 cigarettes per day], medium-intensity smoker [10-19 cigarettes per day], high-intensity smoker [20 or more cigarettes per day])

Supplementary table 3. Risk of chronic back pain and back-related disability at follow-up in 2017-19 associated with changes in sleep quality from 1995-97 to 2006-08 and adherence to physical activity recommendations in 2006-08. Main analysis repeated with additional adjustment for physical work demands and shift work.

	Meeting physical activity guidelines				Not meeting physical activity guidelines			
Risk of back pain and change in sleep quality from 1995-97 to 2006-08	No. of persons	No. of cases	Age-adjusted, RR[a]	Multi-adjusted RR (95% CI)[b]	No. of persons	No. of cases	Age-adjusted, RR[a]	Multi-adjusted RR (95% CI)[b]
Chronic back pain								
Remained good sleep	3,486	636	1.00	1.00 (reference)	3,004	568	1.08	1.01 (0.91-1.13)
Poor sleep to good sleep	207	50	1.35	1.32 (1.00-1.75)	270	66	1.36	1.20 (0.92-1.56)
Good sleep to poor sleep	557	130	1.30	1.27 (1.06-1.52)	614	146	1.44	1.31 (1.10-1.55)
Remained poor sleep	208	56	1.57	1.46 (1.14-1.87)	255	75	1.88	1.66 (1.35-2.04)
Back-related disability								
Remained good sleep	3,486	242	1.00	1.00 (reference)	3,004	239	1.19	1.11 (0.92-1.34)
Poor sleep to good sleep	207	23	1.71	1.69 (1.11-2.60)	270	34	1.86	1.59 (1.08-2.32)
Good sleep to poor sleep	557	62	1.63	1.57 (1.18-2.08)	614	69	1.81	1.60 (1.22-2.10)
Remained poor sleep	208	25	1.79	1.63 (1.06-2.51)	255	39	2.35	2.01 (1.40-2.89)

Abbreviations: CI, confidence interval; RR, risk ratio

[a]Adjusted for age (continuous)

[b]Adjusted for age (continuous), sex (women, men), education (primary school, high school, college ≤4 years, college >4 years), body mass index (continuous), relative change in body weight (continuous), smoking (never smoked, former smoker, current low-intensity smoker [<10 cigarettes per day], medium-intensity smoker [10-19 cigarettes per day], high-intensity smoker [20 or more cigarettes per day]), physical work demands (mostly sedentary, much walking, much walking or lifting, heavy physical work), and shift work (yes, no)

Supplementary table 4. Risk of chronic back pain and back-related disability at follow-up in 2017-19 associated with changes in sleep quality from 1995-97 to 2006-08 and adherence to physical activity recommendations in 2006-08. Main analysis repeated with additional adjustment for comorbid condition(s).

	Meeting physical activity guidelines				Not meeting physical activity guidelines			
Risk of back pain and change in sleep quality from 1995-97 to 2006-08	No. of persons	No. of cases	Age-adjusted, RR[a]	Multi-adjusted RR (95% CI)[b]	No. of persons	No. of cases	Age-adjusted, RR[a]	Multi-adjusted RR (95% CI)[b]
Chronic back pain								
Remained good sleep	3,486	636	1.00	1.00 (reference)	3,004	568	1.08	1.01 (0.90-1.12)
Poor sleep to good sleep	207	50	1.35	1.30 (0.98-1.71)	270	66	1.36	1.19 (0.91-1.55)
Good sleep to poor sleep	557	130	1.30	1.27 (1.06-1.51)	614	146	1.44	1.26 (1.06-1.50)
Remained poor sleep	208	56	1.57	1.47 (1.15-1.88)	255	75	1.88	1.65 (1.34-2.01)
Back-related disability								
Remained good sleep	3,486	242	1.00	1.00 (reference)	3,004	239	1.19	1.10 (0.91-1.33)
Poor sleep to good sleep	207	23	1.71	1.65 (1.08-2.54)	270	34	1.86	1.57 (1.07-2.31)
Good sleep to poor sleep	557	62	1.63	1.56 (1.18-2.07)	614	69	1.81	1.52 (1.16-2.00)
Remained poor sleep	208	25	1.79	1.64 (1.07-2.51)	255	39	2.35	1.98 (1.38-2.84)

Abbreviations: CI, confidence interval; RR, risk ratio

[a]Adjusted for age (continuous)

[b]Adjusted for age (continuous), sex (women, men), education (primary school, high school, college ≤4 years, college >4 years), body mass index (continuous), relative change in body weight (continuous), smoking (never smoked, former smoker, current low-intensity smoker [<10 cigarettes per day], medium-intensity smoker [10-19 cigarettes per day], high-intensity smoker [20 or more cigarettes per day]), and comorbid condition(s) (yes, no)

Supplementary table 5. Risk of chronic back pain and back-related disability at follow-up in 2017-19 associated with changes in sleep quality from 1995-97 to 2006-08 and adherence to physical activity recommendations in 2006-08. Main analysis repeated with additional adjustment for anxiety and/or depression.

	Meeting physical activity guidelines				Not meeting physical activity guidelines			
Risk of back pain and change in sleep quality from 1995-97 to 2006-08	No. of persons	No. of cases	Age-adjusted, RR[a]	Multi-adjusted RR (95% CI)[b]	No. of persons	No. of cases	Age-adjusted, RR[a]	Multi-adjusted RR (95% CI)[b]
Chronic back pain								
Remained good sleep	3,486	636	1.00	1.00 (reference)	3,004	568	1.08	1.00 (0.90-1.12)
Poor sleep to good sleep	207	50	1.35	1.30 (0.99-1.72)	270	66	1.36	1.19 (0.92-1.55)
Good sleep to poor sleep	557	130	1.30	1.23 (1.03-1.47)	614	146	1.44	1.26 (1.06-1.50)
Remained poor sleep	208	56	1.57	1.44 (1.12-1.84)	255	75	1.88	1.57 (1.27-1.94)
Back-related disability								
Remained good sleep	3,486	242	1.00	1.00 (reference)	3,004	239	1.19	1.10 (0.91-1.33)
Poor sleep to good sleep	207	23	1.71	1.67 (1.09-2.56)	270	34	1.86	1.59 (1.08-2.33)
Good sleep to poor sleep	557	62	1.63	1.53 (1.15-2.03)	614	69	1.81	1.55 (1.18-2.05)
Remained poor sleep	208	25	1.79	1.65 (1.07-2.52)	255	39	2.35	1.94 (1.33-2.82)

Abbreviations: CI, confidence interval; RR, risk ratio

[a]Adjusted for age (continuous)

[b]Adjusted for age (continuous), sex (women, men), education (primary school, high school, college ≤4 years, college >4 years), body mass index (continuous), relative change in body weight (continuous), smoking (never smoked, former smoker, current low-intensity smoker [<10 cigarettes per day], medium-intensity smoker [10-19 cigarettes per day], high-intensity smoker [20 or more cigarettes per day]), and anxiety and/or depression (yes, no)
